# Experimental evolution reveals adaptive pathways to reduced antibiotic susceptibility in Pseudomonas aeruginosa biofilms

**DOI:** 10.1099/mic.0.001715

**Published:** 2026-05-28

**Authors:** Fauve Vergauwe, Andrea Sass, Abhinav Madduri, Filip Van Nieuwerburgh, Tom Coenye

**Affiliations:** 1 Laboratory of Pharmaceutical Microbiology, Ghent University, Ghent, Belgium; 2 Laboratory of Pharmaceutical Biotechnology, Ghent University, Ghent, Belgium

**Keywords:** biofilm, experimental evolution, *Pseudomonas aeruginosa*

## Abstract

In the present study, we used an experimental evolution approach to investigate how reduced antibiotic susceptibility develops in biofilms formed by *Pseudomonas aeruginosa* when they are exposed to the clinically relevant antibiotics tobramycin and meropenem. Two biofilm model systems were used, one mimicking surface-attached biofilms (multiple independent *P. aeruginosa* lineages originating from *P. aeruginosa* AA2-1 and maintained for 4–10 cycles) and one mimicking biofilm aggregates in cystic fibrosis sputum (multiple independent *P. aeruginosa* lineages originating from 6 different reference strains and maintained for 15 cycles). Whole-genome sequencing of evolved populations revealed both known and previously unknown genetic adaptations that reduce antibiotic susceptibility. While reduced susceptibility to meropenem commonly arose from mutations in *oprD*, we also identified a role for *irlR*. The development of reduced susceptibility towards tobramycin was more complex, with mutations in *fusA1*, *anr*, *ccoQ1*, *ptsP*, *phoQ* and *nppA1A2BCD* associated with adaptation to this antibiotic. Our findings underscore the multifaceted strategies employed by *P. aeruginosa* to withstand antibiotic pressure in biofilms.

## Data availability

The raw reads generated in this study are available in the ArrayExpress database under the accession numbers E-MTAB-12091 and E-MTAB-11894.

## Introduction

*Pseudomonas aeruginosa* is a versatile opportunistic pathogen that presents a clinical challenge due to its high antibiotic resistance; it is intrinsically resistant to multiple antibiotics due to low outer membrane permeability, production of the AmpC *β*-lactamase and constitutive expression of the MexAB-OprM efflux pump system [1,3]. Its large genome (5.5–7 Mb) provides considerable genetic flexibility, enabling adaptation to fluctuating environmental conditions, including antibiotic exposure [3,4]. Chronic *P. aeruginosa* infections (e.g. respiratory tract infections in people with cystic fibrosis; pwCF) are frequently treated with antibiotics such as tobramycin and meropenem [5]. Tobramycin, an aminoglycoside, often administered via inhalation, disrupts protein synthesis by binding to the A-site of the 30S ribosomal subunit, leading to translational errors and the production of nonfunctional or toxic proteins. To counteract the antimicrobial activity of aminoglycosides, *P. aeruginosa* employs several mechanisms, including the production of aminoglycoside-modifying enzymes, methylation of the 16S rRNA to alter the drug target site [6] and upregulation of the MexXY-OprM efflux pump (the latter often mediated by mutations in the MexZ repressor) [7]. Meropenem, a carbapenem, inhibits cell wall synthesis by binding to penicillin-binding proteins. Resistance to meropenem commonly involves mutations that lead to reduced activity or loss of the OprD porin, reducing antibiotic uptake [1,8]. Besides these known resistance mechanisms, the presence of a protective biofilm matrix (limiting antibiotic penetration into the biofilm) and altered metabolic activity in biofilms further complicate antimicrobial treatment [9,10]. However, many mechanisms of reduced antimicrobial susceptibility – especially those relevant in biofilm conditions – remain poorly understood, and elucidating these mechanisms is essential in order to increase our fundamental understanding of biofilm-associated resistance and tolerance.

In the present study, we performed experimental evolution studies in which *P. aeruginosa* biofilms were cultivated under controlled conditions [11]. Two different setups were used, allowing us to investigate (i) the adaptation of surface-attached *P. aeruginosa* biofilms to tobramycin and meropenem [12] and (ii) the adaptation of suspended *P. aeruginosa* biofilm aggregates formed in the *in vivo-*like synthetic cystic fibrosis medium (SCFM2) to tobramycin [13].

## Methods

### Bacterial strains, culture conditions and chemicals

The bacterial strains used in this study include *P. aeruginosa* AA2-1 (a *lasR*-positive isolate derived from the AA2 strain) (accession number in the BCCM/LMG Bacteria Collection: LMG 27630), LESB58 (LMG 27622), LES431 (LMG 27624), UCBPP-PA14 (LMG 27639), IST27 (LMG 27643) and CF1 (CF isolate Denmark, ST 560) [14,15]. Strains obtained through experimental evolution are listed in Table S1 (available in the online Supplementary Material).

Bacteria were stored at −80 °C in 8% DMSO or using Microbank vials (Pro Lab Diagnostics) and were cultured at 37 °C on Tryptone Soy Agar (TSA; Neogen) plates, in Tryptone Soy Broth (TSB; Neogen), Brain-Heart Infusion Broth (BHI; Neogen), Mueller-Hinton Broth (MH; Neogen) or SCFM2 that was prepared as described previously [16], with the modification that mucin was sterilized by autoclaving instead of UV exposure. The following antibiotics were used: tobramycin, ceftazidime and aztreonam (all from TCI Europe), meropenem (Fresenius Kabi), amikacin disulphate salt, gentamicin sulphate salt, colistin sulphate salt, imipenem, piperacillin sodium salt and cefotaxime sodium salt (all from Sigma-Aldrich). Stock solutions of 10 mg ml^−1^ were prepared in MilliQ water and filter-sterilized (PES, 0.22 µm, VWR).

### Experimental evolution in a bead-based biofilm model

Experimental evolution was performed using the previously described bead-based biofilm model [12] in which surface-attached biofilms form on Microbank beads (Fig. S1). Six independent lineages of *P. aeruginosa* AA2-1 were allowed to evolve in the presence or absence of tobramycin or meropenem. Microbank beads were first rinsed with 0.9% (w/v) NaCl and subsequently vortexed; this was repeated three times to remove remains of the cryopreservative. One millilitre of fresh inoculum containing ~5×10^7^ c.f.u. ml^−1^ was added to seven beads in a well of a 24-well microtiter plate. After 24 h of static biofilm formation at 37 °C, supernatant was removed, and the beads were rinsed three times with 0.9% NaCl. Next, 1 ml BHI was added to the untreated control beads, and 1 ml antibiotics (3 µg ml^−1^ tobramycin or meropenem) dissolved in BHI were added to the other beads. The antibiotic concentrations were selected based on preliminary experiments; we aimed for concentrations that lead to a significant reduction in cell numbers compared to the untreated control, but not complete eradication, so that regrowth in the following cycles can occur. After 24 h of static incubation at 37 °C, the supernatant was removed, and the beads were rinsed three times with 0.9% NaCl. Two beads were collected for storage at −80 °C by transferring them to a cryovial containing BHI+8% DMSO. Four other beads were transferred to a Falcon tube containing 8 ml BHI, which underwent three cycles of vortexing (1 min) and sonication (1 min, 40 kHz, Branson 3510, Branson Ultrasonics) to remove the biofilm cells from the beads. Next, 6 ml of the bacterial suspension was transferred to a fresh Falcon tube, where antibiotics (tobramycin or meropenem) were added to the treated samples at a concentration of 0.5 µg ml^−1^. Planktonic cells were allowed to regrow at 37 °C for 24 h with shaking at 250 r.p.m. The remaining 2 ml of bacterial suspension was used for quantification of the number of surviving cells by plating on TSA. After regrowth of the planktonic cells, fresh beads in a 24-well microtitre plate were inoculated with 5×10^7^ c.f.u. ml^−1^ to start a new cycle.

### SCFM2 model

In the SCFM2 evolution model, biofilms formed by six different *P. aeruginosa* strains were allowed to evolve for 15 cycles, with or without exposure to the antibiotic tobramycin [17] (Fig. S2). For each strain, 4 control lineages and 4 tobramycin-treated lineages were included (except for CF1; 3 each), resulting in 46 independently evolved lineages in total. Overnight cultures of *P. aeruginosa* were diluted in SCFM2 to ~5×10^7^ c.f.u. ml^−1^. Then, 100 µl of the bacterial suspension was added to a flat-bottom 96-well plate and incubated for 24 h at 37 °C under static, aerobic conditions. The following day, biofilms were treated with 100 µl of tobramycin solution (in SCFM2) or 100 µl of SCFM2 medium (for untreated controls). Tobramycin concentrations were selected based on preliminary experiments that resulted in ~1 log (i.e. 90%) reduction in c.f.u./ml of a 24 h old biofilm: 16, 32, 64 or 128 µg ml^−1^ for strains CF1, UCBPP-PA14 and IST27, AA2-1 and LES431 and LESB58, respectively. After an additional 24 h incubation at 37 °C, biofilm aggregates were disrupted by shaking (5 min, 900 r.p.m.) and sonication (5 min). Next, the number of c.f.u./ml after each cycle was quantified by plating dilutions on TSA plates. A frozen stock of each lineage was stored at −80 °C in cryovials with 8% DMSO in TSB. An overnight culture was prepared by inoculating 5 ml TSB with 30 µl of the disrupted biofilm suspension and incubating for 24 h at 37 °C while shaking. The next day, a new cycle was initiated by inoculating with 5×10^7^ c.f.u. ml^−1^ from that overnight culture. This process was repeated for 15 cycles.

### Selecting isolates

To investigate the impact of specific mutations occurring at 100% frequency in a genetically homogenous background, we selected single-colony isolates originating from these evolved strains (Table S1). The selection of mutations to be investigated further depended on whether (i) they showed a strong correlation with reduced antibiotic susceptibility, (ii) they consistently appeared in lineages evolved in the presence of an antibiotic and (iii) they were already known to be involved in reduced susceptibility. First, the evolved populations containing the mutations of interest were plated on agar supplemented with antibiotics, allowing the selection of single colonies capable of growth under these conditions. Specifically, TSA plates with 2 or 4 µg ml^−1^ tobramycin were used for selecting the AA2-1 TOB-01–TOB-20 isolates or CF1 isolates 11–20, respectively, and TSA plates with 1 µg ml^−1^ meropenem were used to select the AA2-1 MER-01–MER-20 isolates. After incubation, single colonies were randomly picked from each plate, with the aim of capturing diverse colony morphologies that likely represent different subpopulations. For each tobramycin- and meropenem-evolved population from the bead-based biofilm model, we selected a total of 20 isolates. From the CF1 populations evolved with tobramycin, we selected ten isolates. These isolates were then characterized to individually confirm the presence of the high-frequency mutations identified in the population-level sequencing and to facilitate further investigation of each mutation’s impact on phenotype.

### Antibiotic susceptibility testing

Minimal inhibitory concentrations (MICs) were determined according to the EUCAST guidelines using the broth microdilution method [18], with the exception that strains from the bead evolution experiment were tested in BHI medium instead of MH. Absorbance was measured at 590 nm with an Envision microplate reader (PerkinElmer). MICs were defined as the lowest concentration of an antimicrobial agent that inhibited at least 90% of microbial growth after 24 h of incubation. The biofilm prevention concentration (BPC) [19] was assessed similarly as described above for the MIC determination, but using serial antibiotic dilutions in SCFM2 medium, which promotes the formation of biofilm aggregates. Bacteria were inoculated at a final concentration of 5×10^7^ c.f.u. ml^−1^ in SCFM2 [20]. After 24 h of incubation at 37 °C, the contents of each well were plated using the microdilution method; after 24 h, colonies were counted, and the number of c.f.u. was determined. The BPC was defined as the lowest antibiotic concentration that limited biofilm formation to a maximum of 10% compared to the untreated control. The minimal biofilm inhibiting concentration (MBIC) was determined in a similar way, except that biofilms were allowed to form for 24 h before antibiotic exposure. Specifically, 50 µl of a 5×10^7^ c.f.u. ml^−1^ bacterial suspension in SCFM2 was added to round-bottom 96-well plates and incubated for 24 h at 37 °C. Next, double-concentrated tobramycin solutions in SCFM2 were added to each well for a further 24 h incubation at 37 °C. Biofilms were then disrupted by vortexing and sonicating the plates for 5 min each, and the well contents were plated on TSA to count the colonies. The MBIC was defined as the lowest antibiotic concentration that resulted in at least 90% reduction of biofilm growth compared to the untreated control. All experiments were performed in biological triplicate, and median values were calculated. Anaerobic MBIC data were collected using a similar set-up, except bacterial suspensions and biofilms treated with tobramycin were incubated in an anaerobic chamber at 37 °C in SCFM2 medium supplemented with 100 mM KNO_3_.

### Growth curves

*P. aeruginosa* overnight cultures were diluted in BHI to a final concentration of 1×10^6^ c.f.u. ml^−1^. Then, 200 µl of this inoculum was transferred to each well of a round-bottom 96-well plate. Growth (in biological duplicates) was monitored either without treatment or with 1 µg ml^−1^ meropenem. The OD at 600 nm was measured every 30 min for at least 24 h at 37 °C using a Victor Nivo plate reader (PerkinElmer). Subsequently, growth curves were analysed to extract key parameters using a combination of numerical methods and logistic model fitting. Carrying capacity (defined as the maximum OD value reached during the growth period, representing the plateau phase of the logistic growth curve), maximum growth rate (μ_max_) (estimated as the steepest slope of the OD vs. time curve, calculated using finite differences between consecutive time points), lag phase duration (defined as the time required for the OD to reach 10% of the carrying capacity, indicating the adaptation period before exponential growth) and area under the curve (AUC; computed using the trapezoidal rule to quantify the total biomass accumulation over time) were calculated for each sample. All analyses were performed using Python 3.12, with the following libraries: NumPy v1.26.4 (for numerical operations), SciPy v1.13.0 (for curve fitting and integration) and Pandas v2.2.2 (for data manipulation).

### DNA extraction

DNA extractions were performed using acid-washed glass beads (Sigma-Aldrich, particle size ≤106 µm). Briefly, overnight broth cultures were centrifuged to obtain a pellet that was resuspended in 200 µl 10 mM TE-buffer (10 mM Tris-HCl pH 8, 1 mM EDTA pH 8), after which 100 µl was transferred to a 2 ml microcentrifuge tube containing ~500 µl acid-washed glass beads and 500 µl lysis buffer (50 mM Tris-HCl pH 8, 70 mM EDTA pH 8, 1% sodium dodecyl sulphate with 0.5 mg ml^−1^ Pronase; Roche). Samples were vortexed vigorously for 5–10 s, incubated at 37 °C for 30–60 min and then centrifuged at 13,000 r.p.m. for a short spin. Following the addition of 200 µl of saturated ammonium acetate, samples were vortexed vigorously in a horizontal position for 5–10 s and centrifuged again at 13,000 r.p.m. for 2 min. Subsequently, 600 µl chloroform was added, and samples were vortexed horizontally for 5–10 s and centrifuged for 5 min at 13,000 r.p.m. Then, 400 µl of clear aqueous top phase was transferred to an Eppendorf LoBind microcentrifuge tube containing 1 ml 100% ethanol. The tubes were mixed by inversion to clot the DNA, followed by centrifugation at 13,000 r.p.m. for 5 min. Afterwards, the supernatant was discarded, and the pellet was washed with 500 µl 70% ethanol. After a short spin, the ethanol was removed by pipetting, and the pellet was air-dried. The dry pellet was dissolved in 300 µl low EDTA TE buffer (10 mM Tris-HCl pH 8, 0.1 mM EDTA, 0.5 µg ml^−1^ RNase) and incubated at 37 °C for 60 min. DNA concentrations were quantified using the BioDrop µLITE (BioDrop).

### Whole-genome sequencing and data analysis

A PCR-free library preparation was performed using the NEBNext Ultra II Library Prep Kit for Illumina, following a size selection protocol using AMPure XP beads after adapter ligation. Samples were sequenced on the Illumina NextSeq 500 System, generating 75 bp single-end reads. The reads were analysed with CLC Genomics Workbench (version 22.0.2) and mapped to reference genomes of *P. aeruginosa* AA2 (NZ_CP051547.1), LESB58 (NC_011770.1), LES431 (NC_023066.1), UCBPP-PA14 (NZ_CP034244.1) or to the 28 contigs of the strain F69A isolate IST27 (whole genome shotgun sequencing project MCMX01000001 to MCMX01000028), or the 36 contigs of CF1. Reads were mapped using a local alignment and filtered based on a 50% length fraction and 80% similarity fraction. The basic variant detection tool was used to detect SNPs with a minimum frequency of 10%, minimum count of 5, minimum quality of 20 and minimum forward/reverse balance of 0.3. All SNPs were manually screened to remove false positives. Insertions and deletions were detected using the InDels and Structural Variants tool, with a minimum sequence complexity of 0.2 and a minimum count of 5. Entries meeting minimum requirements were further manually filtered to remove false positives derived from sequencing and mapping errors. The raw reads generated in this study are available in the ArrayExpress database under the accession numbers E-MTAB-12091 and E-MTAB-11894.

### Quantitative PCR

Gene expression levels of *oprD*, *arnB*, *armZ* and *mexX* were quantified using quantitative PCR (qPCR). Briefly, 25 ml of BHI medium was inoculated with 1 ml of an overnight culture at an OD of 1.0. After 2 h of incubation at 37 °C and 100 r.p.m., when the culture reached an OD of 0.3–0.4, untreated cells were harvested as controls. To assess gene expression upon antibiotic exposure, 1 µg ml^−1^ meropenem or 4 µg ml^−1^ tobramycin was added at OD 0.3–0.4, and incubation continued for an additional 30 min until the OD reached 0.5–0.6. Cultures were then placed on ice, and 4 ml of each sample was transferred into two chilled Eppendorf tubes. Cells were pelleted by centrifugation at 14,000 r.p.m. for 1 min at 4 °C. The supernatant was discarded, and the cell pellets were stored at −80 °C until further processing. Total RNA was extracted using TRIzol reagent (Ambion), followed by DNase treatment and RNA cleanup. RNA concentrations were determined with a BioDrop µLITE spectrophotometer, and cDNA was synthesized using the High Capacity cDNA Reverse Transcription Kit (Applied Biosystems). qPCR was performed using a CFX96 Real-Time System C1000 thermal cycler (Bio-Rad), with the GoTaq qPCR Master Mix (Promega). The primers used are listed in Table S4. The cycling programme began with an initial denaturation step at 95 °C for 3 min, followed by 50 cycles of denaturation (15 s at 95 °C), annealing (30 s at 62 °C for *rpoD*, *oprD* and *armZ*, or 66 °C for *arnB* and *mexX*) and extension (15 s at 72 °C). A melting curve was then performed from 60°C to 98 °C in 0.5 °C increments, measuring fluorescence at each step. Cq values were normalized to the stably expressed reference gene *rpoD* (average Cq value across conditions tested: 19.18, sd: 0.45, *n*=15). Experiments were carried out in biological duplicates (each consisting of technical replicates).

### Statistical analysis

Statistical analysis was performed using IBM SPSS Statistics (version 29). The normal distribution of data was verified with a Shapiro–Wilk test. If the data were normally distributed, an independent samples t-test was performed to compare differences between two timepoints or two treatment groups. If the data were not normally distributed, a nonparametric Mann–Whitney U test was performed. The qPCR data were analysed using one-way ANOVA with post-hoc Tukey analysis. Parameters derived from growth curves were analysed using one-way ANOVA with Bonferroni correction for multiple tests. Graphs were constructed using GraphPad Prism (version 10.6.0).

## Results and discussion

### Experimental evolution in a bead-based biofilm model

**Fig. 1.**
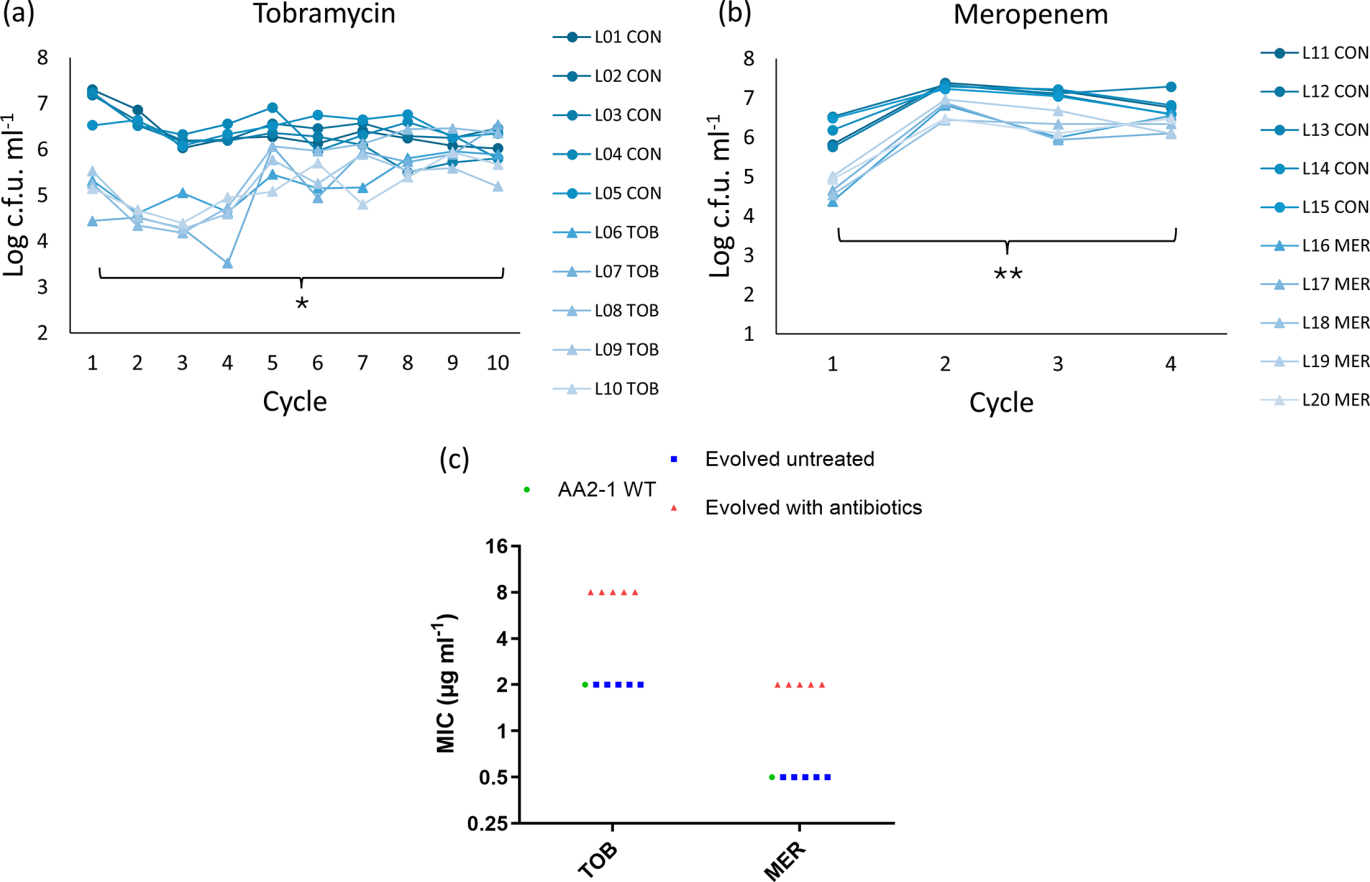
Number of c.f.u. ml^-1^ recovered after each cycle during experimental evolution with the bead-based evolution model for (**a**) untreated (L01-L05 CON) and tobramycin-treated lineages (L06-L10 TOB) and (**b**) untreated (L11-L15 CON) and meropenem-treated lineages (L15-L20 MER) (*P<0.05, **P<0.01). (**c**) Minimal inhibitory concentrations (MIC) of tobramycin (TOB) and meropenem (MER) for *P. aeruginosa* AA2-1 WT, evolved untreated lineages (L01-L05 CON, L11-L15 CON) and lineages that either evolved with tobramycin (L06-10 TOB) or meropenem (L15-L20 MER). Individual datapoints represent the median value of three biological replicates.

In this model, *P. aeruginosa* AA2-1 biofilms were grown on the surface of beads in BHI medium and repeatedly exposed to tobramycin or meropenem or left untreated. For each condition, five independent lineages were included. In a first experiment, untreated and tobramycin-treated lineages were maintained for ten cycles. After ten cycles, the tobramycin-treated lineages showed a significantly higher number of c.f.u. compared to the first cycle, suggesting the occurrence of adaptations that reduced susceptibility to the antibiotic (Fig. 1). In a second experiment, lineages were exposed to meropenem. Already after four cycles, we observed significantly higher c.f.u. counts compared to the first cycle, indicating rapid adaptation to meropenem (Fig. 1); because of this, the experiment was stopped after these four cycles. While the MICs of tobramycin and meropenem for the evolved untreated lineages were identical to the ones of the unevolved WT, lineages exposed to an antibiotic during evolution showed increased MICs (Fig. 1).

To investigate the underlying cause of reduced susceptibility, whole genome sequences (WGS) were determined for all evolved lineages in order to identify all mutations (Table S2). The experimentally evolved populations likely contain multiple subpopulations with different mutations occurring at varying frequencies. After initially characterizing the entire evolved population, we sought to explore the impact of specific mutations in more detail. To this end, we isolated single colonies from the evolved populations by plating them on agar supplemented with antibiotics, selecting for the most resistant colonies. These isolates were sequenced and phenotypically characterized.

### Mutations in *oprD* lead to a reduced meropenem and imipenem susceptibility, while mutations in *irlR* do not reduce *oprD* expression

**Fig. 2.**
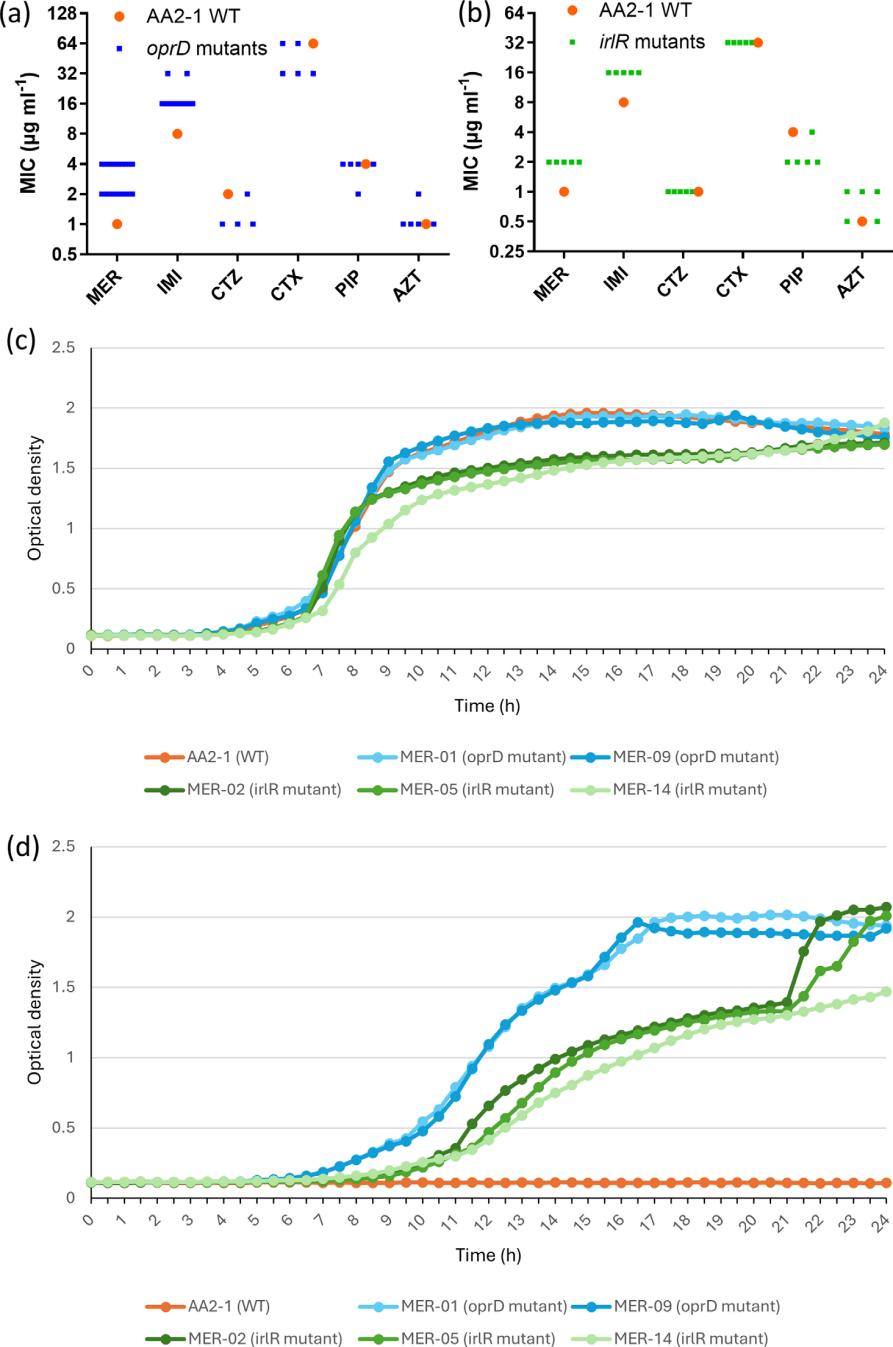
(**a**) Minimal inhibitory concentration (MIC, µg ml^-1^) of meropenem (MER), imipenem (IMI), ceftazidime (CTZ), cefotaxime (CTX), piperacillin (PIP) and aztreonam (AZT) for *P. aeruginosa* isolates evolved in the presence of meropenem. Orange dots represent the WT strain AA2-1, while blue squares represent *oprD* mutant strains (MER-01, MER-03, MER-07–MER-13, MER-15, MER-16 and MER-18–MER-23). (**b**) MIC of MER, IMI, CTZ, CTX, PIP and AZT for *P. aeruginosa* isolates evolved in the presence of meropenem. Orange dots represent the WT strain AA2-1, while green squares represent strains with *irlR* mutations (MER-02, MER-05, MER-06, MER-14 and MER-17). Each datapoint represents the median value of three biological replicates. (**c, d**) Representative growth curves of *P. aeruginosa* AA2-1 WT, *oprD* mutants (MER-01 and MER-09) and *irlR* mutants (MER-02, MER-05 and MER-14) in the absence (**c**) or presence (**d**) of 1 µg ml^−1^ meropenem.

In surface-attached *P. aeruginosa* biofilms that were repeatedly exposed to 3 µg ml^−1^ meropenem, mutations in *oprD* occurred in all lineages. WGS revealed a range of SNPs, insertions and deletions in *oprD*; in total, ten distinct *oprD* mutations occurred: six caused frameshift mutations, three were large deletions and one introduced a premature stop codon. Some *oprD* mutations appeared alone, while others co-occurred with mutations in *lasR*, *rpoS* or *qslA* (Table S5). All of these changes are hypothesized to result in a loss of OprD function. *oprD* encodes for a substrate-specific porin located in the outer membrane of *P. aeruginosa*, where it mainly facilitates the uptake of basic amino acids and small peptides. Importantly, OprD also serves as the principal entry route for carbapenems, with loops 2 and 3 of the porin being especially important for this function [21]. Consequently, mutations that downregulate or inactivate *oprD* reduce the entry of carbapenems into the bacterial cell and confer reduced susceptibility, and such *oprD* mutations are frequently found in carbapenem-resistant *P. aeruginosa* isolates recovered from pwCF [8,22, 23]. Antibiotic susceptibility testing against various *β*-lactams showed that *oprD* mutants exhibited a reduced susceptibility to the carbapenems meropenem and imipenem, with a two- or fourfold increase in MICs, while MIC values for other *β*-lactams tested (ceftazidime, cefotaxime, piperacillin and aztreonam) remained unchanged (Fig. 2). This finding is in line with other data that indicate OprD is a carbapenem-specific porin [24].

Isolates evolved in the presence of meropenem that did not acquire a mutation in *oprD* acquired mutations in *irlR*. The two-component system (TCS) response regulator *irlR* has not previously been implicated in meropenem resistance, and to date, its function remains elusive. All *irlR* mutations resulted in the amino acid substitution Asp185Gly, and they consistently co-occurred with mutations in genes coding for the transcriptional regulators *pqsR* and *lasR*, the chemoreceptor protein *wspA* and sometimes with mutations in genes encoding the response regulators *ladS* or *gacA*, or in HHA37_07410 (coding for a protein of unknown function, belonging to the family of DUF4349 domain-containing proteins) or PA1201 (Tables S2 and 5). All isolates carrying an *irlR* mutation showed a twofold increase in MIC of meropenem and imipenem (Fig. 2). Several isolates showed a twofold decrease in MIC of piperacillin. No increase in MIC was observed for ceftazidime, cefotaxime or piperacillin compared to the WT strain, while some isolates showed a twofold increase in the MIC of aztreonam. Although the MIC increase for the carbapenems is modest, it does suggest a low-level effect of the *irlR* mutation on carbapenem susceptibility. It is however important to note that these effects cannot be conclusively attributed to *irlR* alone, because the isolates also contain other mutations.

When the growth curves of AA2-1 WT were compared with those of *oprD* mutants (MER-01 and MER-09) and *irlR* mutants (MER-02, MER-05 and MER-14), *irlR* mutants showed a growth defect compared to WT and *oprD* mutants in the absence of meropenem [significantly lower µ_max_ than *oprD* mutants (*P*<0.001); significantly lower AUC than WT (*P*=0.02) and *oprD* mutants (*P*=0.004)] (Fig. 2). When exposed to 1 µg ml^−1^ meropenem, the WT is unable to grow, while *oprD* and *irlR* mutants are capable of growing, albeit that both sets of mutants have a significantly lower µ_max_ (*P*<0.01) and AUC (*P*<0.001) and a significantly longer lag phase (*P*<0.001) compared to when grown without meropenem (Fig. S3). The observed differences suggest that *oprD* and – in particular – *irlR* mutations affect bacterial fitness in the absence of meropenem, although the presence of additional mutations in these isolates prevents attributing the slower growth solely to mutations in *oprD* or *irlR*.

We subsequently hypothesized that *irlR* mutations influence *oprD* expression, thereby contributing to carbapenem resistance. El Khoury *et al*. reported that imipenem-resistant *P. aeruginosa* mutants typically harbour a mutation either in *oprD* or in a TCS [25], and certain TCSs are known to regulate *oprD* expression [26,27]. To test whether *irlR* regulates *oprD* expression, *oprD* gene expression in the *irlR* mutant isolate MER-05 was compared with that of AA2-1 WT using qPCR. However, *oprD* expression remained unchanged in MER-05, and meropenem exposure did not affect *oprD* expression (Fig. S4); the mechanism by which the *irlR* mutation contributes to carbapenem resistance remains to be identified.

### Mutations in *ccoQ1*, *ptsP* and *fusA1* contribute to reduced tobramycin susceptibility in surface-attached *P. aeruginosa* biofilms

**Fig. 3.**
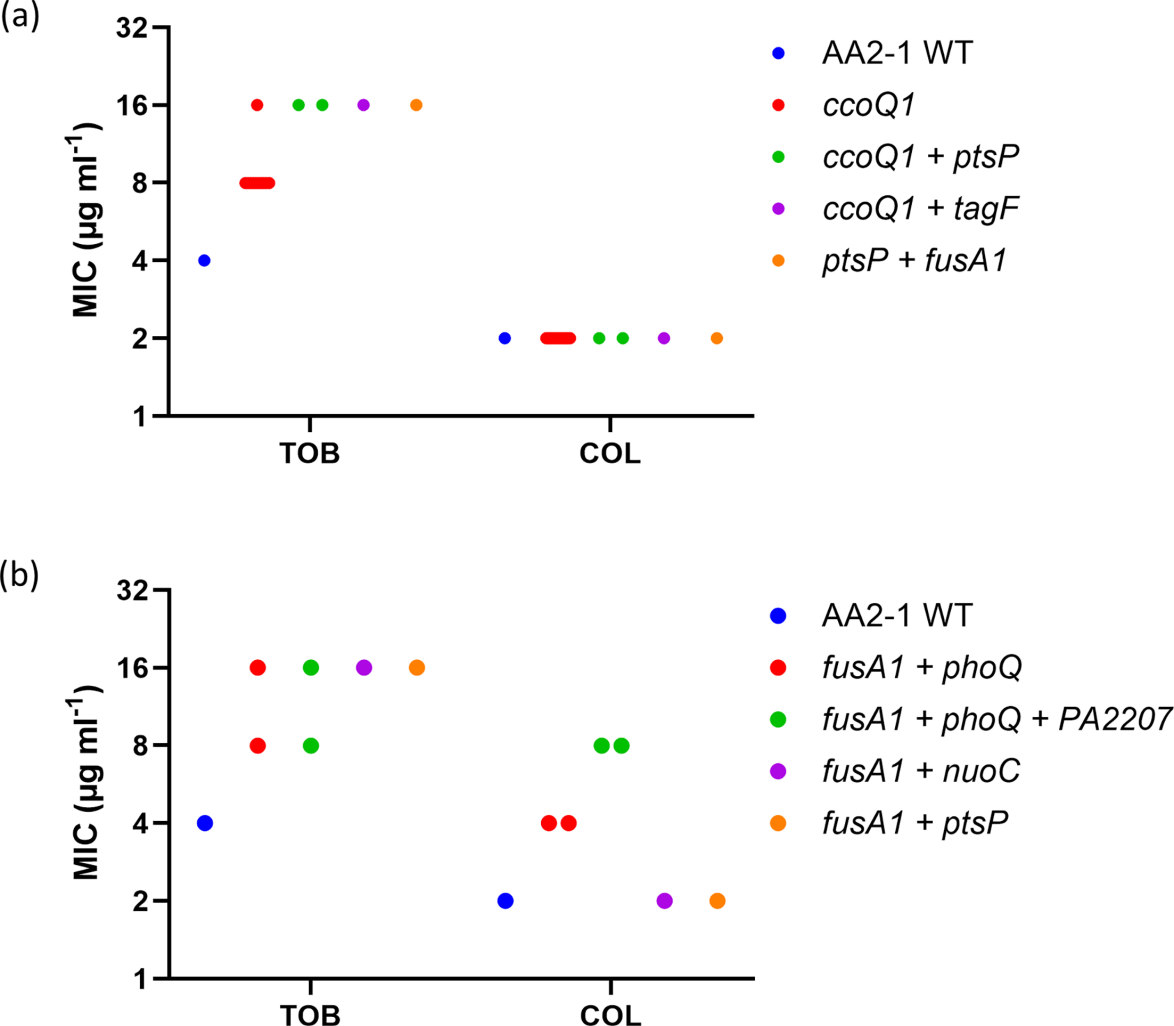
Antibiotic susceptibility of *P. aeruginosa* isolates evolved in the presence of tobramycin. (**a**) Minimal inhibitory concentration (MIC, µg m^l-1^) of tobramycin (TOB) and colistin (COL). Blue: WT strain AA2-1; red: *ccoQ1* mutants (TOB-01–TOB-12); green: *ccoQ1+ptsP* mutants (TOB-10 and TOB-13); purple: *ccoQ1+tagF* mutant (TOB-14); orange: *ptsP+fusA1* mutant (TOB-18). (**b**) MIC of TOB and COL. Blue: WT strain AA2-1; red: *fusA1+phoQ* mutants (TOB-15 and TOB-17); green: *fusA1+phoQ*+ PA2207 mutants (TOB-16 and TOB-19); purple: *fusA1+nuoC* mutant (TOB-20); red: *fusA1+ptsP* mutant (TOB-18).

Multiple isolates that evolved in the presence of tobramycin acquired a Gly8Arg mutation in CcoQ1 (Table S6). These *ccoQ1* mutants exhibited a twofold increase in tobramycin MIC compared to the WT (Fig. 3). CcoQ1 is a subunit of a cbb_3_-type cytochrome c oxidase (cbb_3_-1) encoded by the *ccoN1O1Q1P1* operon. This cbb_3_ oxidase has a high affinity for oxygen, is constitutively expressed across a range of oxygen concentrations and is the dominant terminal oxidase at oxygen concentrations of 20% and (to a lesser extent) 2% [28,29]. Although the exact role of cytochromes in aminoglycoside activity in *P. aeruginosa* is not fully understood, it is established that aminoglycoside transport across the cytoplasmic membrane is an energy-dependent process which relies on the electron transport chain. Therefore, we hypothesize that an impaired respiration in *ccoQ1* mutants leads to a decreased membrane potential and, as a consequence, to a reduced aminoglycoside uptake [30]. In contrast, colistin uptake is not energy-dependent, explaining why colistin susceptibility remained unchanged in isolates with *ccoQ1* mutations (Fig. 3). Furthermore, a shift towards a metabolically less active state could promote antibiotic tolerance [10]. To confirm this mechanism, future experimental work should study tobramycin uptake in *ccoQ1* mutants under biofilm conditions.

Two substitutions in PtsP (Gly676Asp and Glu677Lys) emerged in isolates evolved in the presence of tobramycin (Table S6). However, the *ptsP* mutations never appeared alone; they were always accompanied by mutations in either *ccoQ1* or *fusA1*. Similarly, also in *P. aeruginosa* PA14, *ptsP* mutations were reported to co-occur with *fusA1* mutations during evolution experiments [31]. All isolates harbouring *ptsP* mutations showed a fourfold increase in tobramycin MIC, with no changes in colistin MIC (Fig. 3), while isolates carrying only *ccoQ1* mutations showed a mere twofold increase in tobramycin MIC, suggesting an additive effect of mutations in *ptsP. ptsP* encodes a phosphoenolpyruvate phosphotransferase that is part of the nitrogen phosphotransferase system. Although mutations in *ptsP* have been linked to tobramycin resistance, the exact mechanism remains unclear [13,30,32]. In *P. aeruginosa*, PtsP has been implicated in biofilm formation [33] and quorum sensing (QS) regulation [34]. The nitrogen phosphotransferase system regulates the TCS GigA/GigB in *Acinetobacter baumannii*, enabling it to regulate gene expression in response to antibiotic exposure [35]. GigA/GigB promote survival in stress conditions, while the nitrogen phosphotransferase system negatively regulates the response. *P. aeruginosa* encodes homologues of *gigA* and *gigB*, i.e. PA2798 and PA2797, respectively, which both are essential for intrinsic aminoglycoside resistance [35,36]. Although the precise mechanism by which mutations in *ptsP* lead to reduced tobramycin susceptibility in *P. aeruginosa* biofilms remains to be elucidated, it may involve a homologue of the GigA/GigB pathway or arise through altered biofilm formation and/or QS regulation.

**Fig. 4.**
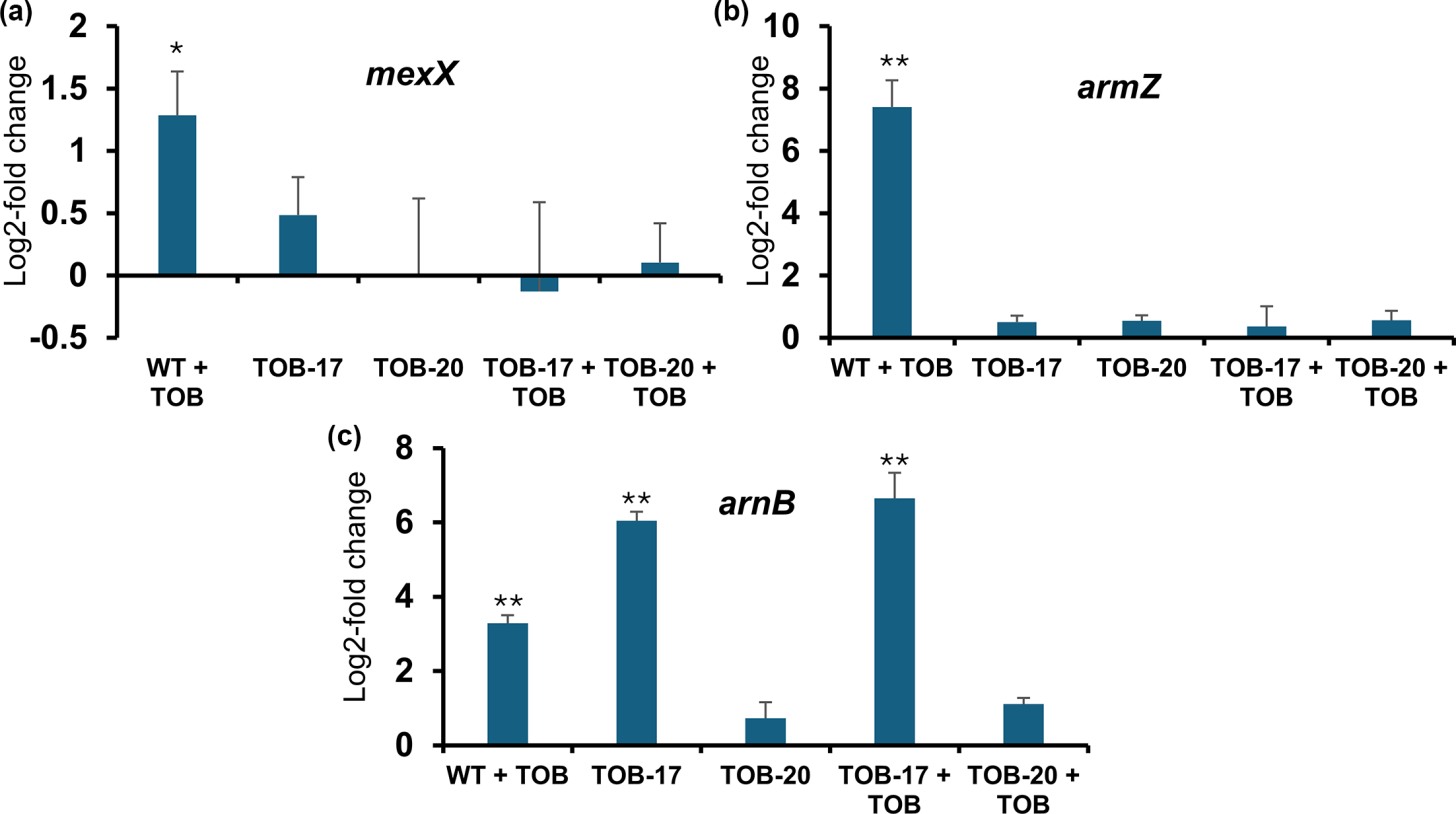
(**a, b**) Effect of *fusA1* mutations in isolate TOB-17 (containing *fusA1+phoQ* mutations) and TOB-20 (containing *fusA1+nuoC* mutations) on *mexX* (**a**) and *armZ* (**b**) expression. (**c**) Effect of *phoQ* mutations in isolate TOB-17 (containing *fusA1+phoQ* mutations) on *arnB* expression. TOB-20 was included as a control strain that contains a mutation in *fusA1*, but not in *phoQ*. Gene expression was determined with and without the addition of 4 µg ml^−1^ tobramycin (TOB). Differences are expressed as log2-fold changes in gene expression compared to the WT strain AA2-1 (that does not contain a *fusA1* mutation) in the absence of tobramycin. Error bars represent sd between duplicates. *P<0.05, **P<0.01.

AA2-1 biofilms exposed to tobramycin during evolution displayed *fusA1* mutations that correlated with increased tobramycin MICs (Fig. 3). Specifically, amino acid substitutions Ala555Gly and Ser591Ala were detected. Notably, these *fusA1* mutations never occurred alone but were always accompanied by mutations in *ptsP*, *phoQ*, PA2207 or *nuoC* (Table S6), complicating the efforts to pinpoint the exact contribution of each gene. Nevertheless, all isolates carrying *fusA1* mutations showed two- or fourfold increased tobramycin MICs (Fig. 3). Recent studies have shown that *fusA1* frequently acquires mutations in *P. aeruginosa* isolates recovered from pwCF, many of which exhibit aminoglycoside resistance [37]. *fusA1* encodes elongation factor G (EF-G1A), a key component of the translational machinery; EF-G1A is essential for protein synthesis by mediating the translocation of mRNA and tRNA through the ribosome and participating in ribosome recycling. Observed genetic changes include point mutations resulting in amino acid substitutions in domains II, III and V of EF-G1A [37]. While the precise mechanism by which *fusA1* mutations confer aminoglycoside resistance remains unclear, aminoglycosides bind to the 30S ribosomal subunit and thereby interfere with tRNA translocation, causing misreading of codons and blocking ribosome recycling. As aminoglycosides are not predicted to bind to EF-G1A directly, the reduced susceptibility is likely an indirect consequence of *fusA1* mutations. One hypothesis is that structural changes in EF-G1A caused by *fusA1* mutations disrupt the interaction of aminoglycosides with the ribosome recycling complex [13]. Maunders *et al*. observed that a specific *fusA1* mutant (Pro443Leu) showed increased expression of the MexXY aminoglycoside efflux pump, likely through upregulation of ArmZ, a transcriptional activator of *mexXY* [38]. As MexXY is a major exporter of aminoglycosides, its upregulation can contribute to reduced tobramycin susceptibility. To investigate whether the Ala555Gly and Ser591Ala substitutions in EF-G1A might affect ArmZ or MexX expression, we used qPCR. However, in strains carrying *fusA1+phoQ* mutations (TOB-17), or *fusA1+nuoC* mutations (TOB-20), we did not observe a statistically significant increase in *mexX* or *armZ* expression compared to the WT. Interestingly, expression of *armZ* and *mexX* was induced in AA2-1 WT upon exposure to tobramycin, but not in the two *fusA1* mutants (Fig. 4), suggesting that either the mechanism of upregulation is disrupted by the *fusA1* mutation or that the tobramycin concentration was too low to induce an effect in isolates with reduced tobramycin susceptibility.

### Mutations in *phoQ* might contribute to tobramycin resistance through increased expression of the *arnB* operon

The amino acid substitution Val260Gly in PhoQ was observed in *P. aeruginosa* AA2-1 isolates evolved in the presence of tobramycin. All isolates carrying this mutation displayed increased MICs for tobramycin and colistin compared to the WT (Fig. 3). However, these *phoQ* mutations never appeared on their own; they always co-occurred with mutations in *fusA1*. Consequently, it remains unclear whether changes in tobramycin susceptibility can be attributed to *phoQ*. In *P. aeruginosa*, the sensor kinase PhoQ forms a TCS with the response regulator PhoP that responds to low Mg^2+^ levels and/or the presence of cationic antimicrobial peptides. Upon activation, PhoQ phosphorylates PhoP, which then upregulates several genes, including those in the *arnBCADTEF* operon. These genes modify the LPS with 4-amino-arabinose, thereby decreasing the net negative charge on the outer membrane and reducing binding and permeation of polymyxins, ultimately conferring resistance [39]. Loss-of-function mutations in *phoQ* are known to contribute to high-level polymyxin resistance in clinical *P. aeruginosa* strains [40]. qPCR confirmed that isolate TOB-17, harbouring both *phoQ* and *fusA1* mutations, shows constitutive overexpression of *arnB* (Fig. 4). TOB-20, an isolate with a mutation in *fusA1*, but not in *phoQ*, showed similar *arnB* expression as the WT, suggesting that the effect observed in TOB-17 is most likely due to the *phoQ* mutation and not the *fusA1* mutation. We hypothesize that increased expression of the *arnBCADTEF* operon by PhoPQ may influence tobramycin susceptibility. While a direct role for PhoPQ in tobramycin resistance has not been described, Macfarlane *et al*. demonstrated that *phoP* and *phoQ* mutants exhibited increased MICs under high [Mg^2+^] conditions for other aminoglycosides, such as streptomycin, kanamycin and amikacin [41]. Chambers and Sauer further showed that *brlR* overexpression downregulates *phoPQ* expression and leads to increased tobramycin MICs in *P. aeruginosa* PAO1 [42].

### Experimental evolution of multiple *P. aeruginosa* isolates in SCFM2 in the presence of tobramycin

In SCFM2, *P. aeruginosa* grows as suspended biofilm aggregates similar to those observed in CF sputum and exhibits *in vivo*-like gene expression profiles as well [13,43], making the SCFM2 model a relevant model for replicating the physicochemical conditions encountered by *P. aeruginosa* when it grows in CF sputum. Using this medium, 46 independent *P. aeruginosa* lineages (originating from 6 different reference strains) were maintained for 15 cycles. Half of these lineages remained untreated, while the other half was exposed to tobramycin at a concentration that reduced c.f.u. counts in 24 h old biofilms by 2–3 logs. An initial description of the results obtained with this experiment was previously reported [17]; key findings are summarized below. Throughout the experiment, c.f.u. counts remained stable in the untreated control lineages. In contrast, the c.f.u. values increased significantly in the tobramycin-exposed lineages compared to the start of the experiment (except for those derived from strain AA2-1). At the end of the evolution experiment, the antibiotic susceptibility of the evolved populations was assessed by determining their MIC and BPC; the lineages exposed to tobramycin displayed higher MIC and BPC values than those evolved without antibiotics, indicating that exposure to tobramycin during evolution reduced susceptibility.

To elucidate the underlying mechanisms, WGS of evolved populations was determined. As described previously, the evolved populations were grown on agar supplemented with tobramycin to select single colonies and investigate the effects of specific mutations, which are described in more detail below.

### Mutations in the ATP-binding cassette transporter NppA1A2BCD only have an impact on tobramycin susceptibility under biofilm conditions

When six *P. aeruginosa* strains were evolved in the presence of tobramycin in SCFM2, mutations in multiple genes of the *nppA1A2BCD* cluster appeared in 20 out of 23 independently evolved lineages (Table S7). *nppA1A2BCD* encodes an ATP-binding cassette (ABC) transporter. Loss-of-function mutations in ABC importers can reduce the entry of antimicrobials, whereas upregulation of ABC exporters can facilitate drug efflux, both leading to a reduced antibiotic susceptibility [44]. In *P. aeruginosa* PA14, the ABC transporter NppA1A2BCD is required for uptake of peptidyl nucleoside antibiotics, and deletion of this transporter (*ΔnppBCD*) increases the MIC for the peptidyl nucleoside antibiotics pacidamycin D, blasticidin S and nikkomycin (but does not alter susceptibility to polymyxin B or colistin) [44]. Cianciulli Sesso *et al*. observed that *nppB*, *nppC* and *nppD* were downregulated in *P. aeruginosa* PA14 when grown in the presence of tobramycin compared to an untreated control in SCFM medium [45]. We hypothesized that inactivating these genes would reduce the uptake of tobramycin and thereby reduce susceptibility. While neither the MIC (2 µg ml^−1^) nor the BPC (8 µg ml^−1^) of tobramycin differed between PA14 WT and a previously generated *ΔnppBCD* knockout mutant [44], the MBIC (determined for 24 h old biofilms) of tobramycin was twofold higher (32 µg ml^−1^) in the mutant than in the WT (16 µg ml^−1^). This suggests that NppA1A2BCD primarily plays a role in tobramycin uptake in more mature biofilms. This observation is in line with results from earlier work showing that another *P. aeruginosa* ABC transporter (PA1874-77) only affects tobramycin susceptibility in biofilms (but not in planktonic conditions), and inactivating PA1876 or PA1877 renders *P. aeruginosa* PAO1 biofilms more susceptible to tobramycin without affecting planktonic cells [46,47]. Moreover, gene expression of PA1874 was approximately tenfold higher in biofilms [47].

### Mutations in the anaerobic response regulator Anr lead to reduced tobramycin susceptibility

**Fig. 5.**
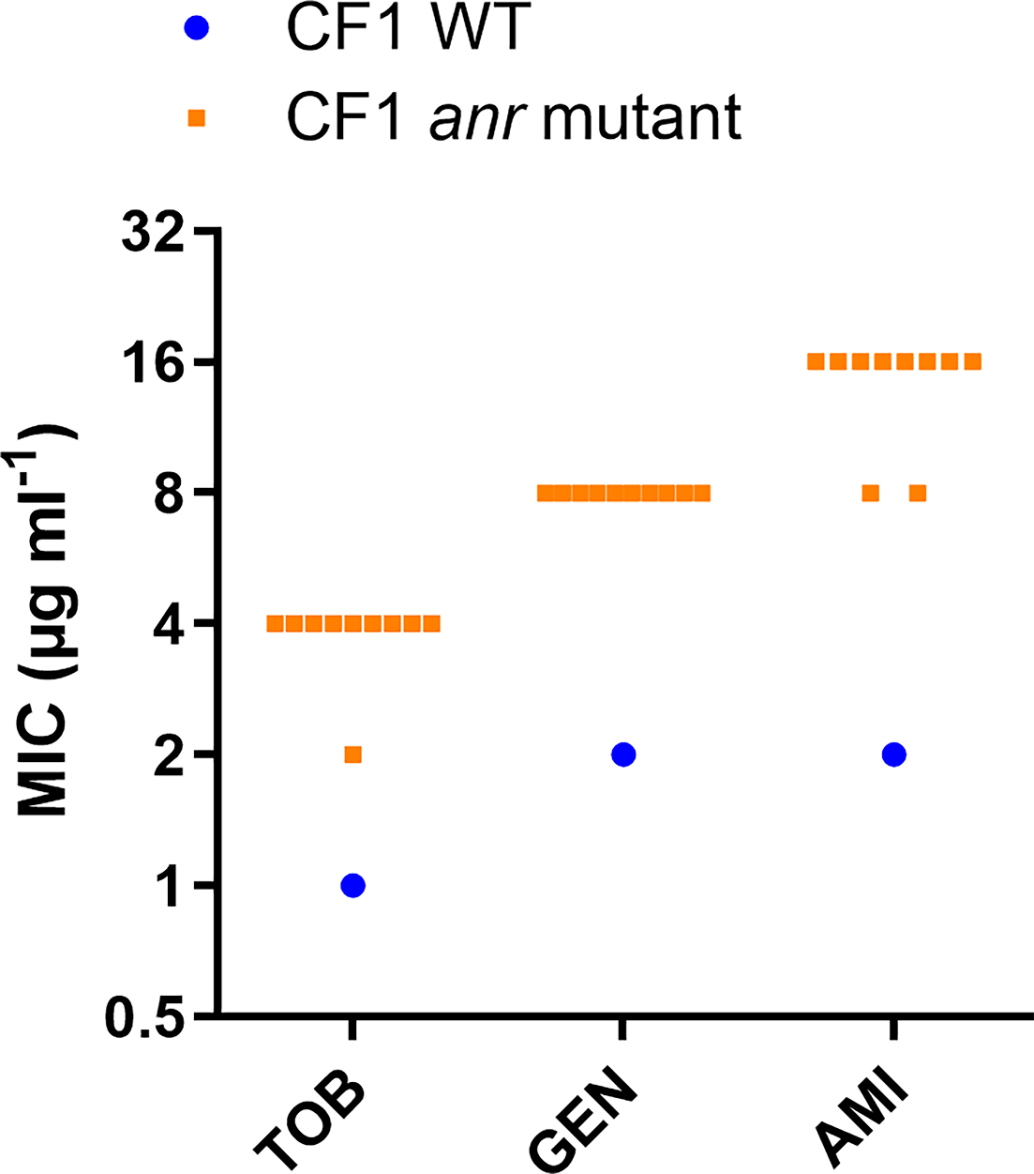
MIC of tobramycin (TOB), gentamicin (GEN) and amikacin (AMI) for CF1 WT and CF1 *anr* mutants (isolates 11–20 containing the Phe107Ser substitution). Each datapoint represents the median value of three biological replicates.

During experimental evolution of *P. aeruginosa* CF1 in SCFM2 in the presence of tobramycin, we identified mutations in the anaerobic transcriptional regulator Anr (Table S3). CF1 isolates carrying the Anr Phe107Ser mutation showed reduced susceptibility to the aminoglycosides tobramycin, gentamicin and amikacin, with two- to fourfold, fourfold and four- to eightfold increases in MIC, respectively (Fig. 5, Table S8).

Anr controls the expression of genes essential for survival under low-oxygen conditions (e.g. genes required for denitrification and anaerobic respiration with nitrate as electron acceptor) by sensing oxygen through a [4Fe-4S] cluster [48,49]. Low-oxygen conditions often predominate in *P. aeruginosa* biofilms, where anaerobic metabolism can significantly influence antibiotic tolerance [10,50, 51]. *anr*-dependent pathways can impact tolerance, especially under these low-oxygen and/or biofilm conditions [52]. Karlowsky *et al*. demonstrated that *anr* mRNA levels were substantially higher in adaptively gentamicin-resistant *P. aeruginosa* than in the WT [52]. This observation aligns with evidence that anaerobically grown *P. aeruginosa* accumulates aminoglycosides less effectively than aerobically grown cells [53]. As aminoglycoside uptake depends on a functional aerobic respiratory pathway [54], upregulation of anaerobic respiration can reduce intracellular aminoglycoside accumulation and thus promote adaptive resistance [52]. However, another study in which an *anr* transposon knockout mutant in PAO1 was used found no difference in tobramycin susceptibility relative to the WT under biofilm conditions [55], and the precise role of Anr in modulating aminoglycoside susceptibility thus remains unclear.

To investigate if mutations in *anr* affect biofilm susceptibility in anaerobic conditions, we determined the MBIC of tobramycin in different conditions (aerobic or anaerobic, with or without nitrate supplementation). When comparing tobramycin MBICs for the CF1-WT strain and two evolved isolates with the Anr Phe107Ser mutation (CF1-16 and CF1-17) in different conditions (Table 1), differences between WT and mutants are modest in aerobic and anaerobic conditions without added nitrate. In conditions with additional nitrate, MBICs are twofold (in aerobic conditions) and two- to fourfold (in anaerobic conditions) higher in the *anr* mutants than in the WT strain, suggesting that the Phe107Ser substitution in Anr provides protection against tobramycin in nitrate-rich conditions.

**Table 1.** Minimum biofilm inhibitory concentration (MBIC) of tobramycin (expressed as µg ml^-1^) obtained under various conditions in SCFM2. Each value is the median of three replicates. Strains CF1-16 and CF1-17 both carry the Anr Phe107Ser mutation

	Aerobic		Anaerobic	
	Without added NO_3_^-^	With added NO_3_^-^	Without added NO_3_^-^	With added NO_3_^-^
**CF1-WT**	8	8	2	8
**CF1-16**	8	16	4	16
**CF1-17**	8	16	2	32

The Phe107Ser substitution is located in the Fe-S cluster-binding domain of Anr, a functionally important region [56]. This domain enables Anr to bind helix-turn-helix motifs in DNA under low-oxygen conditions, thereby activating genes involved in the anaerobic respiratory pathway. Substituting the bulky, hydrophobic phenylalanine with the smaller, polar serine could alter protein stability and/or conformation and may introduce new hydrogen-bonding interactions. Although the Fe-S cluster typically binds the -SH group in cysteine residues, rather than forming hydrogen bonds, structural changes near the cluster-binding region could alter its activity and thereby affect Anr-dependent regulatory pathways critical for biofilm tolerance under anaerobic conditions.

Combined, our observations and literature data suggest that mutations in *anr* can influence a network of genes that govern aminoglycoside susceptibility under the low-oxygen and nitrate-rich conditions typically found in *P. aeruginosa* biofilms.

### Comparison of the mutational landscape between different models and strains

When comparing mutations observed in *P. aeruginosa* AA2 evolved in the two different model systems, it immediately becomes obvious that there is very little overlap (Table S9). Of the 23 mutated genes detected in AA2 populations that were treated with TOB or left untreated, 13 genes only accumulated mutations during evolution in the bead model, and 8 genes only accumulated mutations during evolution in SCFM2. Only two were found in both models, i.e. PA1430 (*lasR*; mutations in this gene were found in both models and in treated as well as untreated lineages) and PA3622 (*rpoS*; mutations in this gene were found in untreated biofilms in the bead model and in biofilms treated with TOB in the SCFM2 model).

Across the entire SCFM2 dataset, a total of 109 genes were found to contain at least 1 mutation. Overall, 24 loci only accumulated mutations after exposure to TOB, 59 loci only accumulated mutations after the control treatment and 18 loci accumulated mutations in both conditions. When comparing different strains evolved in SCFM2, substantial differences in the number of mutated loci were observed, ranging from 5 (CF1) to 50 (PA14) (Fig. S5). For all but one strain (LESB58), more loci accumulated mutations in the untreated controls than when exposed to TOB (Fig. S5). A subset of loci that acquired mutations when exposed to TOB in multiple distinct strains could be identified; these include PA1430, PA1807, PA1808, PA1809, PA4601 and PA5017.

## Conclusion

Experimental evolution is a valuable tool for identifying mechanisms involved in adaptation to antimicrobial agents in *P. aeruginosa* biofilms, as it enables direct comparisons between evolved strains and their ancestor, potentially uncovering new pathways leading to reduced antibiotic susceptibility.

We confirmed that *oprD* mutations are commonly associated with reduced meropenem susceptibility in *P. aeruginosa* biofilms and observed that mutations in the TCS response regulator *irlR* also reduce meropenem susceptibility, although the precise mechanism remains to be elucidated. In the case of tobramycin, the situation is more complex, with mutations in *ccoQ1*, *ptsP*, *fusA1* and *phoQ* contributing to reduced tobramycin susceptibility, although the exact mechanisms are not yet fully understood. We identified a potential role for the ABC transporter NppA1A2BCD in tobramycin uptake in biofilm conditions. The anaerobic response regulator Anr clearly influences susceptibility towards tobramycin, yet additional research is needed to elucidate the underlying mechanisms. In many cases, reduced susceptibility is due to a combination of mutations, highlighting the need for a holistic view on biofilm susceptibility.

Finally, our research highlights that the mode of biofilm growth (e.g. surface-attached vs. suspended biofilms), as well as strain background, can have a profound effect on the outcome of experimental evolution studies, both in terms of phenotype (i.e. antimicrobial susceptibility) and genotype (i.e. which mutations occur). This reinforces the notion that the microenvironment, as well as the genetic background, determines biofilm antimicrobial susceptibility [11,57,60] and that extrapolations between strains and model systems should be approached with care.

A limitation of the present work is that the exact contribution of specific (combinations of) mutations to antibiotic susceptibility could not be confirmed. One way to resolve this could be to complement individual mutations and investigate the antimicrobial susceptibility of the complemented strains, but this was beyond the scope of the present study. Secondly, our experimental setup required a planktonic re-growth phase, in order to have a sufficiently large number of c.f.u. to inoculate the next round of the evolution experiment. The impact of this planktonic re-growth phase on the number and type of mutations identified is currently unknown.

## Supplementary material

10.1099/mic.0.001715 Supplementary Material 1.

10.1099/mic.0.001715 Supplementary Material 2.

## Abbreviations

ABC: ATP-binding cassette
AUC: area under the curve
BHI: Brain-Heart Infusion Broth
BPC: biofilm prevention concentration
MBIC: minimal biofilm inhibiting concentration
MH: Mueller-Hinton Broth
MICs: minimal inhibitory concentrations
qPCR: quantitative PCR
QS: quorum sensing
TCS: two-component system
TSA: Tryptone Soy Agar
TSB: Tryptone Soy Broth
