# Supplementary material for: Experimental evolution reveals adaptive pathways to reduced antibiotic susceptibility in Pseudomonas aeruginosa biofilms
Source: Microbiology (Reading). 2026 May 28;172(5):001715. doi: 10.1099/mic.0.001715 (PMC13218686; doi:10.1099/mic.0.001715)
Supplement: Supplementary Material 1. [file mic-172-01715-s001.pdf]

# Experimental evolution reveals adaptive pathways to reduced antibiotic susceptibility in *Pseudomonas aeruginosa* biofilms

**Fauve Vergauwe[1], Andrea Sass[1], Abhinav Madduri[1], Filip Van Nieuwerburgh[2], Tom Coenye[1]**

## Supplementary information

## Excel files:

**Table S1.** Bacterial strains used in this study.

**Table S2.** All mutations identified in the bead-based biofilm model (population sequencing).

**Table S3.** All mutations observed in SCFM2 model (population sequencing).

**Table S9.** Overview of all *P. aeruginosa* genes in which mutations were observed during the experimental evolution studies.

**Table S4.** qPCR primers used in this study.

| Gene | Forward primer | Reverse primer |
|---|---|---|
| *rpoD* | 5’-GGGCGAAGAAGGAAATGGTC-3’ | 5’-CAGGTGGCGTAGGTGGAGAA-3’ |
| *oprD* | 5’-TCCGCAGGTAGCACTCAGTTC-3’ | 5’-AAGCCGGATTCATAGGTGGTG-3’ |
| *mexX* | 5’-TGTACGAGGAAGGCCAGGAC-3’ | 5’-CTTGATCAGGTCGGCGTAGC-3’ |
| *arnB* | 5’-GAACCAGGAGCTCGAACAGC-3’ | 5’-AGCAGGGTGATGACGTTGG-3’ |
| *armZ* | 5’-TCCTGCAAGAACAGGTCAGC-3’ | 5’-CCAGGTTGTGGTTGATGTCG-3’ |

**Table S5.** Mutations detected in *P. aeruginosa* AA2-1 experimentally evolved in the presence of meropenem, and antibiotic susceptibility data. Abbreviations: Deletion (Del), Insertion (Ins), frameshift mutations (fs), single nucleotide variant (SNV), meropenem (MER), imipenem (IMI), ceftazidime (CTZ), cefotaxime (CTX), piperacillin (PIP) and aztreonam (AZT). * SNVs predicted to occur in noncoding intergenic regions located down- or upstream of coding sequences.

| | PA0958 | PA4885 | PA1003 | PA1430 | PA3974 | PA3708 | PA2586 | | PA3622 | PA1244 | | | MIC (µg/mL, BHI) | | | | | |
|---|---|---|---|---|---|---|---|---|---|---|---|---|---|---|---|---|---|---|
| **Isolate** | ***oprD*** | ***irlR*** | ***pqsR*** | ***lasR*** | ***ladS*** | ***wspA*** | ***gacA*** | ***HHA37_07410*** | ***rpoS*** | ***qslA*** | ***HHA37_21540*** | ***HHA37_19455*** | **MER** | **IMI** | **CTZ** | **CTX** | **PIP** | **AZT** |
| MER-21 | 1148 bp Del | | | | | | | | | | | | 2 | 16 | | | | |
| MER-01 | 131 bp Del | | | | | | | | | | | | 4 | 16 | | | | |
| MER-03 | 131 bp Del | | | | | | | | | | | | 4 | 16 | | | | |
| MER-19 | 312 bp Del | | | | | | | | | | | | 4 | 16 | | | | |
| MER-20 | 312 bp Del | | | | | | | | | | | | 4 | 16 | | | | |
| MER-22 | M111fs (Del) | | | | | | | | | | | | 2 | 16 | | | | |
| MER-09 | M135fs (Del) | | | | | | | | | | | | 4 | 32 | | | | |
| MER-23 | W277* | | | | | | | | | | | | 2 | 16 | | | | |
| MER-13 | Y379fs (Del) | | | | | | | | | SNV upstream of *qlsA* * | | | 4 | 16 | | | | |
| MER-15 | Y379fs (Del) | | | | | | | | | SNV upstream of *qlsA* * | Ins transposase IS3 | | 4 | 16 | | | | |
| MER-02 | | N185G | 12 bp Del | V221M | N545S | V453L | | | | | | | 2 | 16 | 1 | 32 | 2 | 1 |
| MER-06 | | N185G | 12 bp Del | V221M | N545S | V453L | | | | | | | 2 | 16 | 1 | 32 | 2 | 0.5 |
| MER-14 | | N185G | 12 bp Del | V221M | | V453L | | | | | | | 2 | 16 | 1 | 32 | 4 | 1 |
| MER-17 | | N185G | 12 bp Del | V221M | | V453L | | | | | | SNV downstream of 3' end * | 2 | 16 | 1 | 32 | 2 | 0.5 |
| MER-05 | | N185G | 12 bp Del | V221M | | V453L | M57I | I51L | | | | | 2 | 16 | 1 | 32 | 2 | 1 |
| MER-18 | 312 bp Del | | | 18 bp Del | | | | | | | | | 4 | 16 | | | | |
| MER-07 | A280fs (Del) | | | Ins transposase upstream | | | | | | | | | 2 | 16 | | | | |
| MER-08 | A280fs (Del) | | | C203R | | | | | | | | | 2 | 16 | | | | |
| MER-11 | G212fs (Del) | | | T178A | | | | | | | | | 2 | 16 | | | | |
| MER-12 | S153fs (Ins) | | | G191S | | | | | | | | | 2 | 16 | | | | |
| MER-10 | M135fs (Del) | | | | | | | | M1? | | | | 4 | 32 | | | | |
| MER-16 | 312 bp Del | | | | | | | | 52 bp Del | | | | 4 | 16 | | | | |
| AA2-1 WT | | | | | | | | | | | | | 1 | 8 | 1 | 32 | 4 | 0.5 |

**Table S6.** Mutations observed in *P. aeruginosa* isolates experimentally evolved in the presence of tobramycin, and minimal inhibitory concentrations (MIC) of tobramycin (TOB) and colistin (COL).

| | PA1552.1 | PA0337 | | PA4266 | PA1180 | | PA2639 | MIC (µg/mL, BHI) | |
|---|---|---|---|---|---|---|---|---|---|
| **Isolate** | ***ccoQ1*** | ***ptsP*** | ***tagF*** | ***fusA1*** | ***phoQ*** | ***PA2207*** | ***nuoC*** | **TOB** | **COL** |
| TOB-01 | G8R | | | | | | | 8 | 2 |
| TOB-02 | G8R | | | | | | | 8 | 2 |
| TOB-03 | G8R | | | | | | | 16 | 2 |
| TOB-04 | G8R | | | | | | | 8 | 2 |
| TOB-05 | G8R | | | | | | | 8 | 2 |
| TOB-06 | G8R | | | | | | | 8 | 2 |
| TOB-07 | G8R | | | | | | | 8 | 2 |
| TOB-08 | G8R | | | | | | | 8 | 2 |
| TOB-09 | G8R | | | | | | | 8 | 2 |
| TOB-10 | G8R | E677K | | | | | | 16 | 2 |
| TOB-11 | G8R | | | | | | | 8 | 2 |
| TOB-12 | G8R | | | | | | | 8 | 2 |
| TOB-13 | G8R | E677K | | | | | | 16 | 2 |
| TOB-14 | G8R | | R168L | | | | | 16 | 2 |
| TOB-15 | | | | S591A | V260G | | | 16 | 4 |
| TOB-16 | | | | S591A | V260G | R235L | | 8 | 8 |
| TOB-17 | | | | S591A | V260G | | | 8 | 4 |
| TOB-18 | | G676D | | S591A | | | | 16 | 2 |
| TOB-19 | | | | S591A | V260G | R235L | | 16 | 8 |
| TOB-20 | | | | S591A | | | W370R | 16 | 2 |
| AA2-1 WT | | | | | | | | 4 | 2 |

**Table S7.** Genetic variants in the *nppA1A2BCD* cluster observed in *P. aeruginosa* isolates experimentally evolved in the presence of tobramycin.

| Strain | *nppA1* | *nppA2* | *nppB* | *nppC* | *nppD* |
|---|---|---|---|---|---|
| **AA2-1** | | | | L150_T151dup<br>G166S<br>W11*<br>A113fs | T326P<br>Q430_R431dup<br>R84ins |
| **LES 431** | | H306_N307del<br>V171fs | | A238fs | T326P<br>L79fs |
| **LES B58** | | Q344* | A258fs | L150_T151dup<br>G124D<br>R10_R19del | S322fs<br>E174* |
| **IST27** | W44* | | R8L | F71S<br>Y190* | Q163_R164dup<br>E243_F367del<br>I383_E386del |
| **UCBPP-PA14** | | | G18D<br>I27del | W308* | T326P<br>P358fs |

**Table S8.** Mutations observed in *P. aeruginosa* CF1 isolates evolved in the presence of tobramycin, and antibiotic susceptibility data. Minimal inhibitory concentrations (MIC) of tobramycin (TOB), gentamicin (GEN) and amikacin (AMI).

| | PA1544 | MIC (µg/mL, MH) | | |
|---|---|---|---|---|
| **Isolate** | ***anr*** | **TOB** | **GEN** | **AMI** |
| isolate 11 | F107S | 4 | 8 | 8 |
| isolate 12 | F107S | 4 | 8 | 16 |
| isolate 13 | F107S | 4 | 8 | 16 |
| isolate 14 | F107S | 2 | 8 | 8 |
| isolate 15 | F107S | 4 | 8 | 16 |
| isolate 16 | F107S | 4 | 8 | 16 |
| isolate 17 | F107S | 4 | 8 | 16 |
| isolate 18 | F107S | 4 | 8 | 16 |
| isolate 19 | F107S | 4 | 8 | 16 |
| isolate 20 | F107S | 4 | 8 | 16 |
| CF1 WT | | 1 | 2 | 2 |

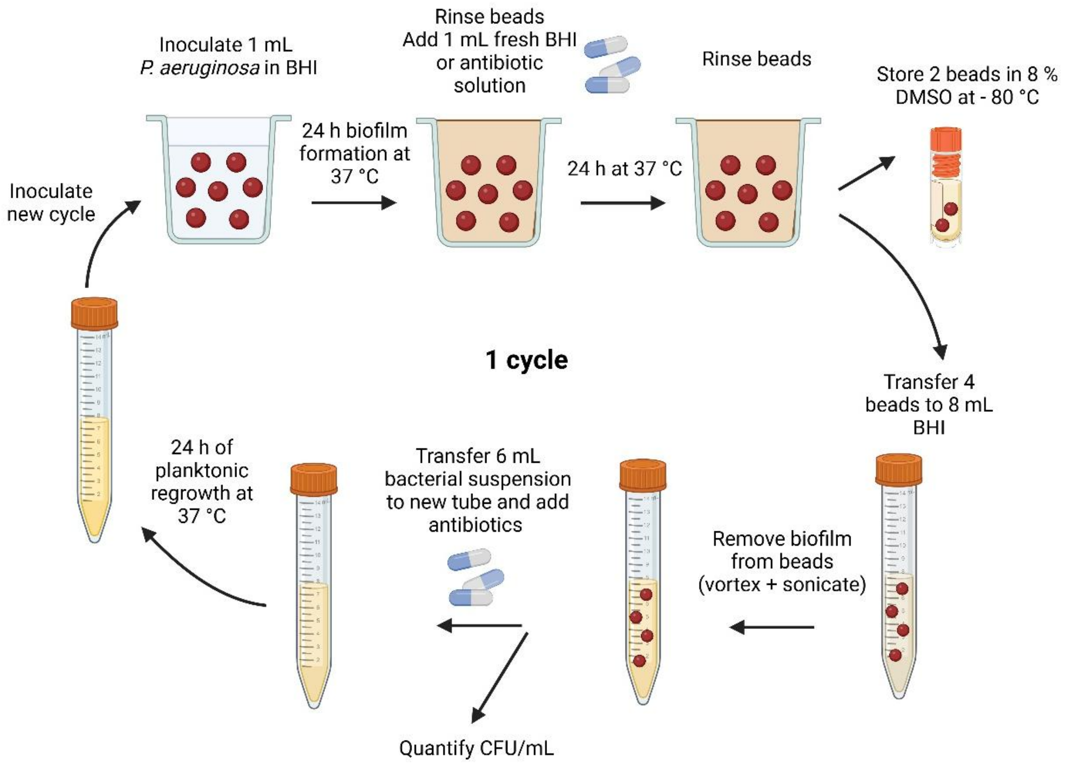


**Fig. S1.** Experimental set-up of the bead-based biofilm evolution model. Fresh inoculum is added to 7 cryobeads (red) in the well of a 24-well microtiter plate. After 24 h, biofilms are formed on the surface of the beads and these are then treated for 24 h. Subsequently, the supernatant is removed and the beads are rinsed. Two beads are stored at -80°C; 4 other beads are transferred to a falcon tube to harvest the sessile cells. A part of these cells is used for quantification, another part is used for planktonic regrowth. Figure created with BioRender.

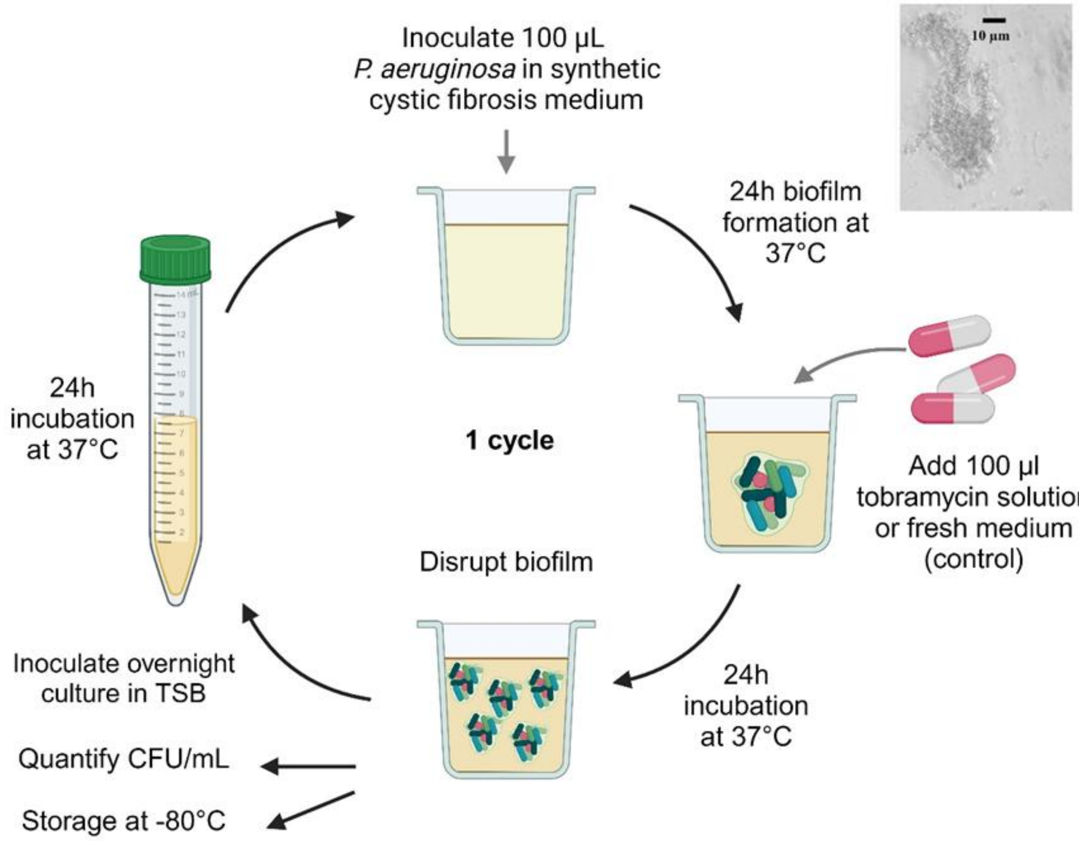


**Fig. S2.** Experimental set-up of the biofilm evolution model in SCFM2. 100 µL of a *P. aeruginosa* culture is added to the well of a 96-well plate. After 24 h, suspended biofilm aggregates are formed and these are either treated with 100 µL of tobramycin, or 100 µL fresh SCFM2 medium is added. After 24 h, the biofilm is disrupted and a part of the bacterial suspension is used to inoculate an overnight culture to allow the start of a new cycle. The number of surviving cells after each cycle is quantified by plating on TSA. The samples are stored at -80 °C. Figure created with BioRender.

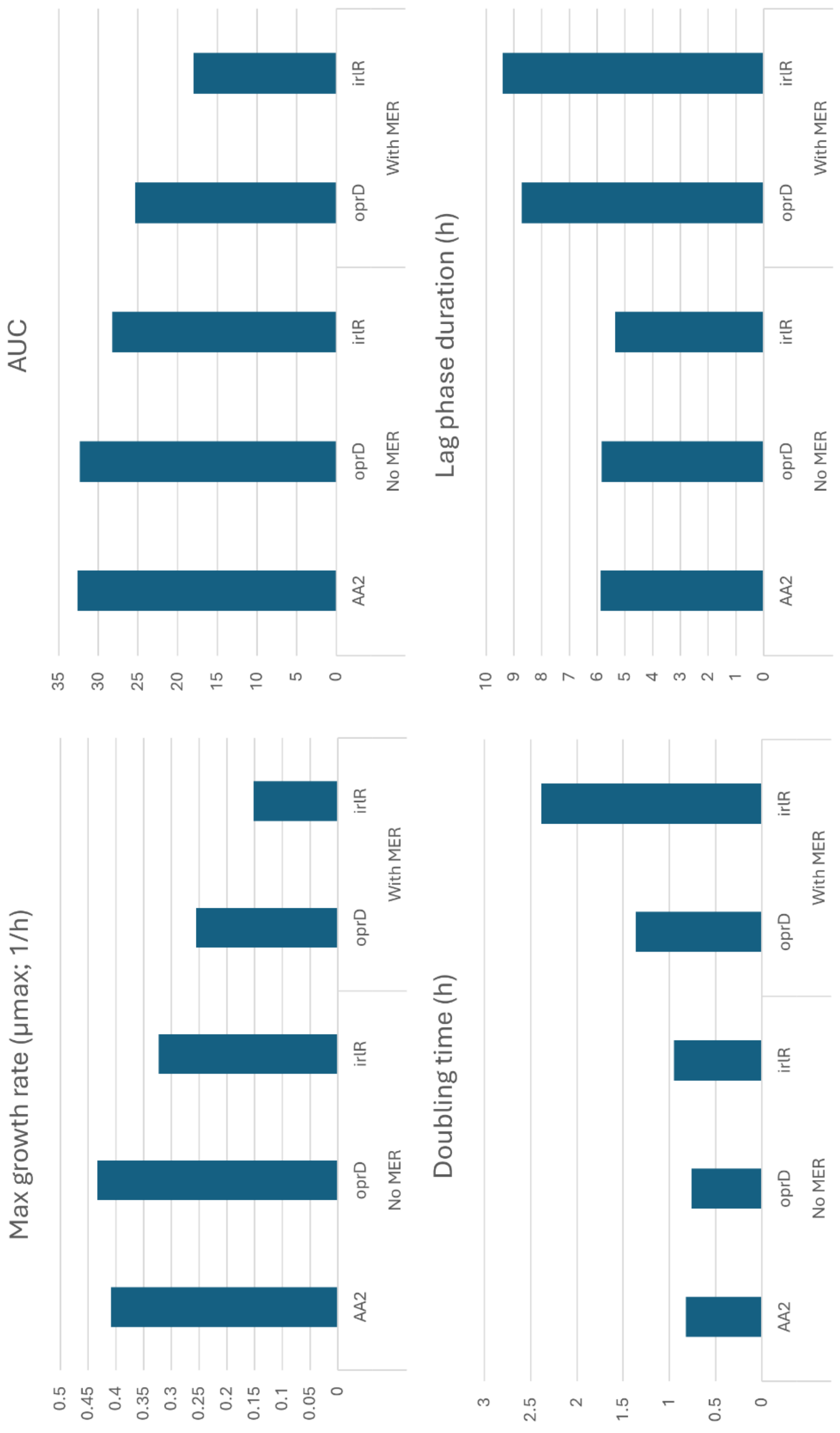


**Fig. S3.** Comparison of selected growth parameters (maximum growth rate, area under the curve, doubling time and lag phase duration) for *P. aeruginosa* AA2 WT and selected mutants, in presence and absence of meropenem (MER).

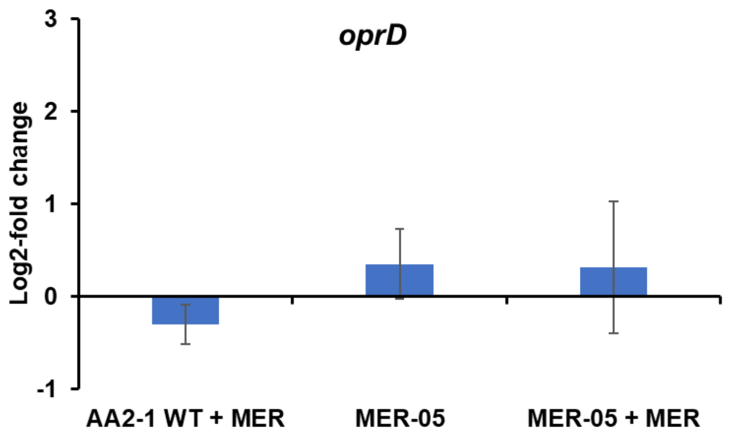


**Fig. S4.** Effect of *irlR* mutation in isolate MER-05 on *oprD* expression, compared to the WT strain AA2-1 that does not contain an *irlR* mutation (as determined by qPCR). The effects are measured with and without addition of 1 µg/mL meropenem (MER). Differences are expressed as log2-fold changes in gene expression. Error bars represent standard deviation between technical replicates.

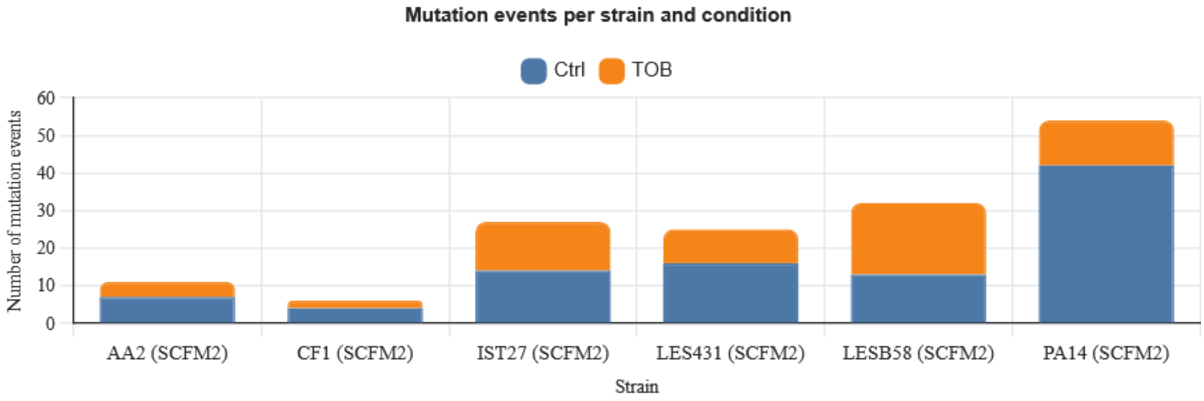


| Strain | Both conditions | Ctrl only (untreated) | TOB only (treated) | Total mutated (union) |
|---|---|---|---|---|
| AA2 | 1 | 6 | 3 | 10 |
| CF1 | 1 | 3 | 1 | 5 |
| IST27 | 4 | 10 | 9 | 23 |
| LES431 | 3 | 13 | 6 | 22 |
| LESB58 | 5 | 8 | 14 | 27 |
| PA14 | 4 | 38 | 8 | 50 |

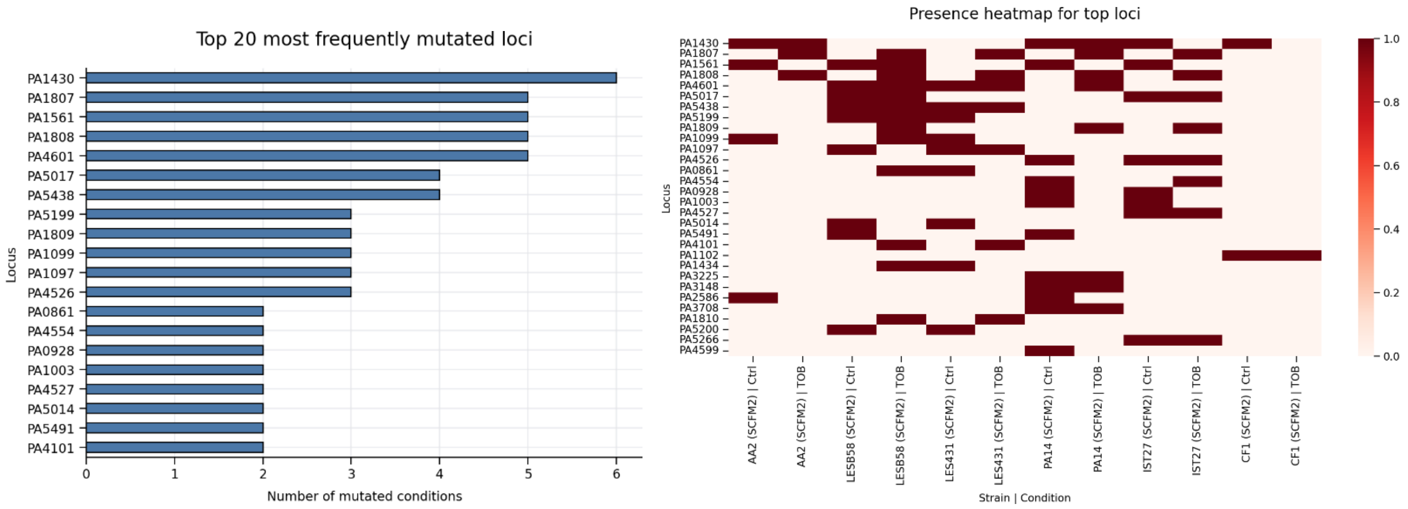


**Fig. S5.** A) Number of mutated genes identified in different strains (top). B) Number of loci mutated in different conditions (middle). C) Number of conditions in which mutations in a certain gene were found (bottom left). D) Distribution of the top 20 most frequently mutated loci over the different strains (bottom right).
